# News and Miscellany

**Published:** 1901-05

**Authors:** 


### News and Miscellany.

**We have received** the catalog of The Summer School of the Medical Department Washington University, St. Louis, Mo. For a copy address R. J. Terry, M. D., Secretary, St. Louis.

**A Lap—a—rot—o—my!** An esteemed correspondent and subscriber suggests that the rotting of the early vegetables in Texas is due to the fact that winter lingered so long in the lap of spring; a kind of lap—a—rotomy, as it were.

**We are indebted** to Lambert Pharmacal Co. for an interesting and instructive little brochure, entitled: "Medical and Surgical Experiences in the South African War," being addresses to the Toronto Clinical Society and Canadian Medical Association by Lieutenant-Colonel G. Sterling Ryerson, M. D., lately British and Canadian Red Cross Commissioner with Lord Robert's headquarters in South Africa. A copy of this book can be had free of charge by sending a postal card to Lambert Pharmacal Co., St. Louis, mentioning the "Red-Back."

NASHVILLE, TENN., April 11, 1901.

At the sixty-eighth annual meeting of the Tennessee Medical Society the following officers were elected:

Deering, J. Roberts, M. D., (Southern Practitioner) Nashville, President; J. B. Murfree, Jr., M. D., Murfreesboro, L. A. Yarbrough, M. D., Covington, W. B. St. John, M. D. Bristol, Vice-Presidents; A. B. Cooke, M. D., Nashville, Secretary; W. C. Bilbro, M. D., Murfreesboro, Treasurer.

Next place of meeting: Memphis, Tenn., on the second Tuesday in April 1902.

A. B. COOKE, M. D., Secretary.

**The State Board of Medical Examiners**—the first Texas ever had—is to be appointed from the list of physicians selected at the meeting and to be submitted to the Governor. Nine are to be appointed from a list of eighteen for the most part able and distinguished men. On the list is a physician from each of the thirteen Congressional Districts and five from the State at large. This doubles up on five of the districts—there being thus, two from each of five districts. From this list the Governor will be able to select nine gentlemen of the highest character,—physicians eminent, able, incorruptible and incorrupt—who will be an honor to the State, as they are an honor to the profession. The list embraces, as will be seen by reference to the report of the meeting published herewith, the president and five ex-presidents of the Association, as follows: Drs. Burroughs, Loggins, Coleman, Wilson, Gardner. The Governor is not required by law, however, to appoint the nine members from nine separate districts. Dr. Jno. C. Jones, from the Gonzales district, was Hood's Chief Surgeon in the Confederate war, a man of unusual ability.

---

**State Medical Association Notes.**—The Association will meet at El Paso next time, 4th Tuesday, April, 1902.

Eighty-one new members were admitted.

The Treasurer has money on hand to throw at the birds.

Miller makes a rattling good Treasurer.

The two pretty young lady stenographers double discounted the clam we had at Waco.

Moore, of Runge, is to deliver the oration next time. The orator didn't show up at Galveston.

The physicians of Galveston were most attentive and courteous. It will be long ere any of us forget them or the Galveston meeting of 1901.

I went out to the college and heard Professor Paine and Professor McLaughlin lecture. The college still shows signs of the terrible assault of September 8.

The sanitary condition of Galveston is excellent, and was a most gratifying surprise to the writer.

I had the honor of meeting the Marine Hospital Service delegate, Dr. C. P. Wertenbaker, the first delegate from that service to the Texas Medical Association. He was sent in response to an invitation embodied in a resolution adopted at Waco, April, 1900. Dr. Wertenbaker is a credit to the Marine Hospital Service. He is an intelligent, well informed and zealous sanitarian and physician, and a delightful companion. Hope to meet him again.

---

### Digitalis.

To a recent number of *The Journal of the American Medical Association* Dr. John Upshur contributes an interesting and instructive article on "Heart Tonics." Of digitalis, he says, that while the primary effect of its administration is slowing of the heart's action, lengthening of diastole and increased arterial tension, its continued exhibition is very apt to be followed by a period of exhaustion, the result of overstimulation of the vagus, cardiac motor ganglia and inhibitory centres. This secondary period of cardiac depresson, of course, usually comes at a time when the heart most needs the drug's sustaining influence. He thinks digitalis is often capable of doing much good, but advises great care in the selection of cases for its use. It is his opinion that in lesions at the aortic orifice the administration of digitalis is apt to do more harm than good, and he does not advise its use even in cases of mitral regurgitation, believing that a period of heart exhaustion will supervene before a compensatory hypertrophy is established. A further contra-indication to the use of this drug is, any tendency to myocardial degeneration; hence he never uses it in the weak heart of fevers, septicemias and the like. He thinks digitalis finds its greatest field of usefulness in the treatment of mitral stenosis, and in lesions at the tricuspid orifice. However, in almost every case in which heart stimulant is indicated he considers strychnia a far safer, and much more reliable agent. Not the least of his reasons for this belief is the very good one, that the use of strychnia is never followed by a secondary period of cardiac exhaustion, as is the case in the use of digitalis.

